# Interactions of Zn(II) Ions with Humic Acids Isolated from Various Type of Soils. Effect of pH, Zn Concentrations and Humic Acids Chemical Properties

**DOI:** 10.1371/journal.pone.0153626

**Published:** 2016-04-14

**Authors:** Patrycja Boguta, Zofia Sokołowska

**Affiliations:** Department of Physical Chemistry of Porous Materials, Institute of Agrophysics, Polish Academy of Sciences, Lublin, Poland; São Paulo State University, BRAZIL

## Abstract

The main aim of this study was the analysis of the interaction between humic acids (HAs) from different soils and Zn(II) ions at wide concentration ranges and at two different pHs, 5 and 7, by using fluorescence and FTIR spectroscopy, as well as potentiometric measurements. The presence of a few areas of HAs structures responsible for Zn(II) complexing was revealed. Complexation at α-sites (low humified structures of low-molecular weight and aromatic polycondensation) and β-sites (weakly humified structures) was stronger at pH 7 than 5. This trend was not observed for γ-sites (structures with linearly-condensed aromatic rings, unsaturated bonds and large molecular weight). The amount of metal complexed at pH5 and 7 by α and γ-structures increased with a decrease in humification and aromaticity of HAs, contrary to β-areas where complexation increased with increasing content of carboxylic groups. The stability of complexes was higher at pH 7 and was the highest for γ-structures. At pH 5, stability decreased with C/N increase for α-areas and -COOH content increase for β-sites; stability increased with humification decrease for γ-structures. The stability of complexes at α and β-areas at pH 7 decreased with a drop in HAs humification. FTIR spectra at pH 5 revealed that the most-humified HAs tended to cause bidentate bridging coordination, while in the case of the least-humified HAs, Zn caused bidentate bridging coordination at low Zn additions and bidentate chelation at the highest Zn concentrations. Low Zn doses at pH 7 caused formation of unidentate complexes while higher Zn doses caused bidentate bridging. Such processes were noticed for HAs characterized by high oxidation degree and high oxygen functional group content; where these were low, HAs displayed bidentate bridging or even bidentate chelation. To summarize, the above studies have showed significant impact of Zn concentration, pH and some properties of HAs on complexation reactions of humic acids with zinc.

## Introduction

Humic acids (HAs)—principal component of humic substances, belong to complex and heterogeneous mixtures of polydispersed dark brown to black organic substances. Their macromolecules are rich in aromatic units, aliphatic chains and functional groups and reveal flexibility and high sensitivity to chemical agents. HAs are formed by humification, which generally involves biochemical processes of decay and transformation of plant and microbial remains. HAs are insoluble under acidic conditions but can be extracted using alkaline solutions. They are some of the most valuable components of the soil environment, mainly due to their significant reactivity. A large sorption capacity in the range of 300–1400 meq 100 g^-1^ makes HAs one of the most charged substances among naturally occurring polyelectrolytes (mineral parts of the soil have from 2 to 30 times lower sorption capacity). Because of this feature, HAs demonstrate a great impact on soil-buffering capacity [1–3] and especially the form, bioavailability and retention abilities of metals in soils [4–7]. They interact with metal ions, hydroxides and oxides on the way of ion-exchange, surface-adsorption, chelation, coagulation, and peptization. Moreover, HAs readily bind clay minerals to form complexes with different properties and stability. Presence of HAs in soil solution also influences availability of important nonmetal elements: N, S and P. High reactivity of HAs has also an impact on transport and degradation of both natural and anthropogenic organic compounds. Such molecules as lignin, its transformation products, polysaccharides, proteins, lipids, nucleic acids remain in continuous equilibrium with fraction of humic substances during mineralization and humification processes. Chelating action of HAs can also cause negative effects e.g.: adsorption of pesticides, phthalates or polycyclic aromatic hydrocarbons. However, interactions of HAs with metal ions are highly complex processes and there are still many unclear, contradictory opinions on the mechanism of their interactions, as well as on the influence of humic acid composition on these interactions [8–11]. The complex structures of HAs are the chief aspect that makes their study difficult. The principal differences in HA structure result from a variable atom composition, e.g., C, O, H and N, which form structures of diverse configurations with various molecular weights, aromatic rings (hydroxyphenol derivatives), heterocycled and condensed rings with heteroatoms, side aliphatic chains and functional groups.

One of the most unclear properties of HAs is related to the type of interaction and the way that HAs bind with metal ions. Studies reveal that carboxylic groups of HAs are mainly responsible for metal binding processes [12]; however there are also reports that indicate complexing with both COOH and OH functional groups [5,13] and activity with OH groups even at low pHs, and the possibility of metal binding with N in rings or amine and amide groups [13,14]. Moreover, detailed studies have revealed that depending on the source material, HAs can display different dissociation constants (pKa) even for the same kind of group (e.g., COOH) [15]. Additional complications are the various properties of each metal and the different kinds of possible interactions, in which ion exchange, complexation, chelation and adsorption with hydroxide formation should be taken into consideration [10,16].

An important aspect of selecting HAs for studies on metal interactions is that these compounds are main components of humic substances and simultaneously demonstrate wide spectrum of behaviour in soil: intermediate between fraction of fulvic acids and humins. Depending on environment conditions, HAs can be found in mobile and well soluble forms (typical for fulvic acids), or as poorly soluble compounds (typical for humins fraction). Similarly, its properties are changed in wide range of e.g. molecular mass, content of functional groups or aromatic and aliphatic units, which also places it between fulvic acids and humins. Such vast diversity of HAs makes them highly interesting group of compounds, which studies can bring some new knowledge on the subject of interactions of natural, organic macromolecules with metals.

Zinc (Zn) is a micronutrient whose presence in soil solution is necessary for plant growth [17]. Soluble forms of Zn-HA complexes probably enable a slow and gradual release of Zn for plant consumption; on the other hand, high concentrations of Zn may have a toxic effect [18] and may pollute the environment even on large scales via water flow [19]. Insoluble Zn-HA compounds can accumulate and lead to soil degradation as well as micronutrient deficiency. The problem of the above interactions is especially important, because the current increase of anthropogenic activities causes elevation of Zn concentrations in aquatic systems. In fact, it has been estimated that there is about twenty times more Zn in some aquatic systems compared to that normally resulting from natural processes [19].

On the basis of the above issues, the main aim of this paper is to study interaction mechanisms of Zn ions with HAs isolated from different soils, focusing on the influence of pH and Zn ion concentration, as well as various chemical properties of HAs such as the content of functional groups, electron donor atoms, aromaticity and degree of humification on interactions with Zn ions.

## Materials and Methods

### Soil material

Soil samples (marked as S1–S5) were collected from the A-horizon of five different soils. S1 sample originated from area periodically flooded by the Wieprz River. The location was covered by grassland vegetation: cl.*Molinio-Arrhenatheretea*, o.*Trifolio fragiferae-Agrosteitalia stoloniferae*. Clearly formed alluvial layers indicated on the soil with young profile. S2 was taken from flat area of the soil exploited as forest with vegetation typical for cl.*Fagetaia sylvaticeae*, ass.*Tilio-Carpinetum* (*Carpinus betulus*, *Quercus robur*, *Corylus avellana*, *Asperula odorata*, *Asarum europaeum*). S3 originated from lowered, expanse and not exploited grassland with plant species characteristic for cl.*Molinio-Arrhenatheretea*, o.*Molinietalia caeruleae*. Sampling of S4 took place in slightly undulating area with loess parent rock of the soil covered by forest with vegetation community typical for cl.*Vaccinio-Piceetea*, ass.*Querco roboris-Pinetum* (*Carpinus betulus*, *Quercus robur*, *Pinus silvestris*, *Fagus silvatica*, *Betula verrucosa*, *Oxalis acetosella*). S5 came from flat region of Kozłowiecki Forest with dominant plant species: cl.*Vaccinio-Piceetea*, ass.*Peucedano Pinetum* (*Pinus silvestris*, *Betula pendula*, *Quercus petraea*). Detailed information concerning physicochemical properties of the soils is presented in Table 1. Soils were air-dried, crushed and passed through a 2 mm sieve. The main properties of the soils were investigated by conventional methods: pH was measured electrochemically in H_2_O and KCl using a digital pH-meter (Radiometer Copenhagen), bulk density (d) was determined by the pycnometric method, porosity (P)—by mercury porosimetry (Autopore IV 9500), ash content (A) was calculated by weighing the residue after 4 h of combustion at 550°C in a muffle furnace (FCF 12 SP, Czylok). Hydrophobic/hydrophylic properties were evaluated by the measurement of contact angles (Θ) [20] using a goniometer (DSA1, Kruss). Total soil carbon (C_tot._) and organic carbon (C_org._) were determined via C/N analyzer (TOC MULTI N/C 2000, Analytik Jena). Cation exchange capacity (CEC) was calculated from potentiometric titration curves as the surface charge at pH 7 [21]. Three replications were performed for each analysis. The results were averaged and are presented in Table 1.

**Table 1:** Physicochemical description of the soil samples.

Soil no	Soil type	Location	pH KCl	pH H_2_O	C_tot._	C_org._	CEC	d	P	A	ϴ
					(%)	(%)	(cmol kg^-1^)	(g cm^-3^)	(%)	(%)	(°)
S1	*Haplic Fluvisol* (Alluvial soil)	51°09’N/22°59’E	5.89	6.56	1.19	0.22	2.51	2.61	47.1	96.2	41.5
S2	*Haplic Chernozem* (Chernozem)	50°32’N/24°01’E	4.32	5.06	1.73	0.50	6.93	2.61	51.1	95.5	39.1
S3	*Mollic Gleysol* (Black Earth)	50°22’N/23°39’E	7.89	7.88	22.0	3.48	45.6	2.09	60.1	64.8	79.7
S4	*Stagnic Luvisol* (Grey-brown soil)	50°38N/22°41’E	5.28	5.94	2.73	0.29	4.07	2.55	53.2	93.3	43.6
S5	*Haplic Cambisol* (Brown Soil)	51°23’N/22°35’E	3.57	4.45	11.4	0.46	3.03	2.57	42.1	95.7	95.7

C_tot._, total carbon; C_org._, organic carbon; CEC, cation exchange capacity; d, bulk density; P, porosity; A, ash; ϴ, contact angle

### Humic acids analyses

The isolation of HAs from soil samples was carried out by alkaline extraction according to the procedure recommended by the International Humic Substances Society [22]. Elemental composition of HAs was measured by using CHN 2400 analyzer (Perkin Elmer). Percent oxygen composition was calculated from the equation %O = 100%-(%H+%C+%N) and H/C, O/H, O/C, and C/N atomic ratios were calculated as well as degree of internal oxidation by Zdanow’s formula: ω = [(2O+3N)-H]/C [23]. The carboxyl groups (COOH) and sum of carboxylic and phenolic groups (COOH+OH) were investigated according to Dragunowa and Kucharenko [24]. The phenol OH groups were evaluated as the difference between the total acidity and carboxyl groups. The E_4_/E_6_ and E_2_/E_6_ parameters were calculated as the ratio of absorbance measured respectively at 465 and 665 nm as well as 280 and 665 nm of HAs (40 mg dm^-3^) in a solution of 0.05M NaHCO_3_ using a UV-VIS spectrometer (Jasco V-520) [25]. The Kumada parameter (ΔlogK) was calculated as the difference between the decimal logarithm of absorbance at 400 and 600 nm: ΔlogK = logA_400_-logA_600_. Three replicates were performed for each treatment and the results were averaged.

### Study of interactions in HA-Zn(II) systems

A stock solution (50 mg dm^-3^) was prepared for each HA by dissolving them in deionized water with small addition of 0.1M NaOH to final pH 8 and under N_2_ atmosphere. A stock solution of Zn(II) (1000 mg dm^-3^) was prepared in deionized water from Zn(II) chloride of analytical grade purity. The pH of the solution was adjusted to 4 with 0.1M HCl.

#### Fluorescence spectroscopy

The stock solution of Zn(II) and deionized water were added in appropriate volumes to aliquots of 40 cm^3^ of the HAs stock solutions to obtain 50 cm^3^ final volume with final HAs concentrations 40 mg dm^-3^ and Zn concentrations ranging from 0 to 50 mg dm^-3^ (0, 2, 4, 6, 8, 10, 15, 20, 30, 40, 50 mg dm^-3^). This series of HA-Zn solutions was prepared for each HA at both pH 5 and 7 using 0.1M HCl/0.1M NaOH. All samples were equilibrated at a constant stirring speed, under a N_2_ atmosphere for 24 h and then the pH was eventually readjusted.

Fluorescence spectra were recorded in emission and excitation mode and as 3D excitation-emission matrices (EEM) using a Hitachi F-7000 FL luminescence spectrometer. Emission spectra were recorded in the range 380–600 nm at a fixed excitation wavelength of 360 nm, whereas excitation spectra were obtained over the scan range of 300 to 500 nm by setting the emission monochromator at 520 nm. EEM spectra were recorded setting emission and excitation slits at 10 nm and selecting a scan speed of 12000 nm min^-1^. The wavelength emission was scanned from 300 to 600 nm, while the excitation wavelength was increased sequentially by 5 nm steps from 250 to 500 nm.

EEM plots were generated as contour maps using the Surfer software (Golden Software Inc., Golden, CO). Fluorescence intensities (FI, arbitrary units) of three fluorescence peaks (designed as α, β and γ), which changed markedly as a function of Zn(II) concentration, were selected to determine the complexing capacities of HAs (C_L_) and the stability constants of HA-Zn(II) complexes (logK) by using the single-site fluorescence quenching model proposed by Ryan and Weber [26].

#### Infrared spectroscopy

To obtain infrared spectra for each HA, solutions of HA-Zn were prepared in a similar way as for fluorescence measurements, i.e. a series of each HA (40 mg dm^-3^) were prepared with selected Zn concentrations (0, 2 5, 10, 50 mg dm^-3^) at both pH 5 and 7. All solutions were lyophilized for 24 h and dried at 105°C and then 1 mg fine-powdered HA-Zn samples were homogenized with 200 mg KBr and analyzed in the form of pellets on a FTIR spectrometer (Tensor 27, Bruker), in the range 400–4000 cm^-1^. High quality of spectra was obtained by 256 scans at 2 cm^-1^ resolution.

#### Proton releasing study

The behavior of HA functional groups under the influence of Zn was investigated potentiometrically by proton-release measurement. Both the HA solution (40 mg dm^-3^), as well as Zn(II) stock solution (1000 mg dm^-3^) were adjusted precisely to pH 5 and 7. Afterwards, all HAs were titrated with Zn(II) (0–50 mg dm^-3^) at constant stirring speed under a N_2_ atmosphere. The pH measurement was recorded after each titration step when the signal from glass electrode was stable.

## Results and Discussion

### Physicochemical properties of soils and humic acids

Principal soil characteristics are summarized in Table 1. The pH measurements revealed that samples S2 and S5 were strongly acidic, S4 acidic, S1 slightly acidic and S3 was an alkaline soil [18]. The total carbon content (C_tot._) varied widely between soils and was the highest for S3 soil, mostly due to the large amount of carbonates (proved by probing the samples with HCl) and was the lowest for alluvial S1 soil because of high amount of sand. The organic carbon content (C_org._) correlated with C_tot._ values and was the highest for S3 and the lowest for S1. The CEC changed in a similar way as the C_org._, which can indicate a dominant role of organic colloids. The density (d) was at similar level for all the samples, however slight differences were observed and, in general, were consistent with the porosity (P) changes. The highest P and the lowest d were measured for S3, mainly due to high C_org._ content. The ash content (A) was similar for almost all studied soils, with the exception of S3. The highest value of the contact angle (ϴ), which was measured for S5, indicated the hydrophobic nature of the studied brown soil surface, while the low ϴ for S1, S2 and S4 revealed the more hydrophilic properties of these soils and, in consequence, their higher abilities to retain water.

Physicochemical properties of isolated HAs are presented in Table 2. The elemental composition indicated some diversity in the content of C, H, N and O of the studied HAs. HA4 demonstrated the highest C content and, simultaneously, the lowest oxygen and nitrogen content, which provides evidence of significant degree of humification. Values of the H/C atomic ratio indicate the presence of aromatic systems conjugated with aliphatic chains (up to 10 C atoms). However, low values of H/C ratios in certain HAs (HA4 or HA1) indicate a great deal of aromaticity and high condensation of aromatic units, while the highest values of the H/C ratios (HA5) can indicate a more significant participation of aliphatic structures. The content of oxygen structures is well-described by the internal oxidation degree (ω), the O/H and especially, O/C ratios (greater variability), with the highest O/C ratio values for HA3 and HA5. HA4 had the highest values of the C/N ratio, which suggests a higher transformation of organic substance with respect to the other HAs. The amount of COOH groups and total of COOH+OH functional groups decreases, respectively, in the order: HA3>HA5>HA1>HA4>HA2 and HA5>HA3>HA1>HA4>HA2, and the values are quite in line with the changes in the values of the O/C atomic ratio. Slight differences can result from the fact that the O/C ratio can also take into account other oxygen-containing groups. The values of E_4_/E_6_ and E_2_/E_6_ ratios decrease in the order: HA3>HA4>HA5>HA1>HA2, which suggests increasing humification degree, molecular mass and presence of condensed aromatic structures in the case of E4/E6 and increasing humification of organic components from early transformation stage like lignins and quinones in the case of E2/E6. The ordering of this sequence has been confirmed by the ΔlogK values, which additionally proves that the sample HA3 is less humified (R-type: slight degree) than all remaining HAs (B-types: weak degree).

**Table 2:** Chemical properties of isolated humic acids.

HAs no	C	H	N	O	H/C	O/H	O/C	C/N	ω	COOH	OH	COOH+OH	E4/E6	E2/E6	ΔlogK
	(atomic %)									(cmol kg^-1^)					
HA1	40.9	35.6	3.27	20.2	0.87	0.57	0.49	12.5	0.36	321	329	650	5.35	28.8	0.70
HA2	40.3	38.0	2.89	18.8	0.94	0.49	0.47	14.0	0.20	196	258	454	4.80	25.1	0.65
HA3	39.4	36.7	2.72	21.2	0.93	0.58	0.54	14.5	0.35	424	246	670	7.08	67.5	0.89
HA4	43.8	35.2	2.13	18.8	0.80	0.53	0.43	20.5	0.20	260	239	499	6.17	41.8	0.75
HA5	36.6	39.4	2.27	21.7	1.08	0.55	0.59	16.2	0.29	411	330	741	5.69	31.6	0.67

### Fluorescence spectroscopy

**Fig 1:**
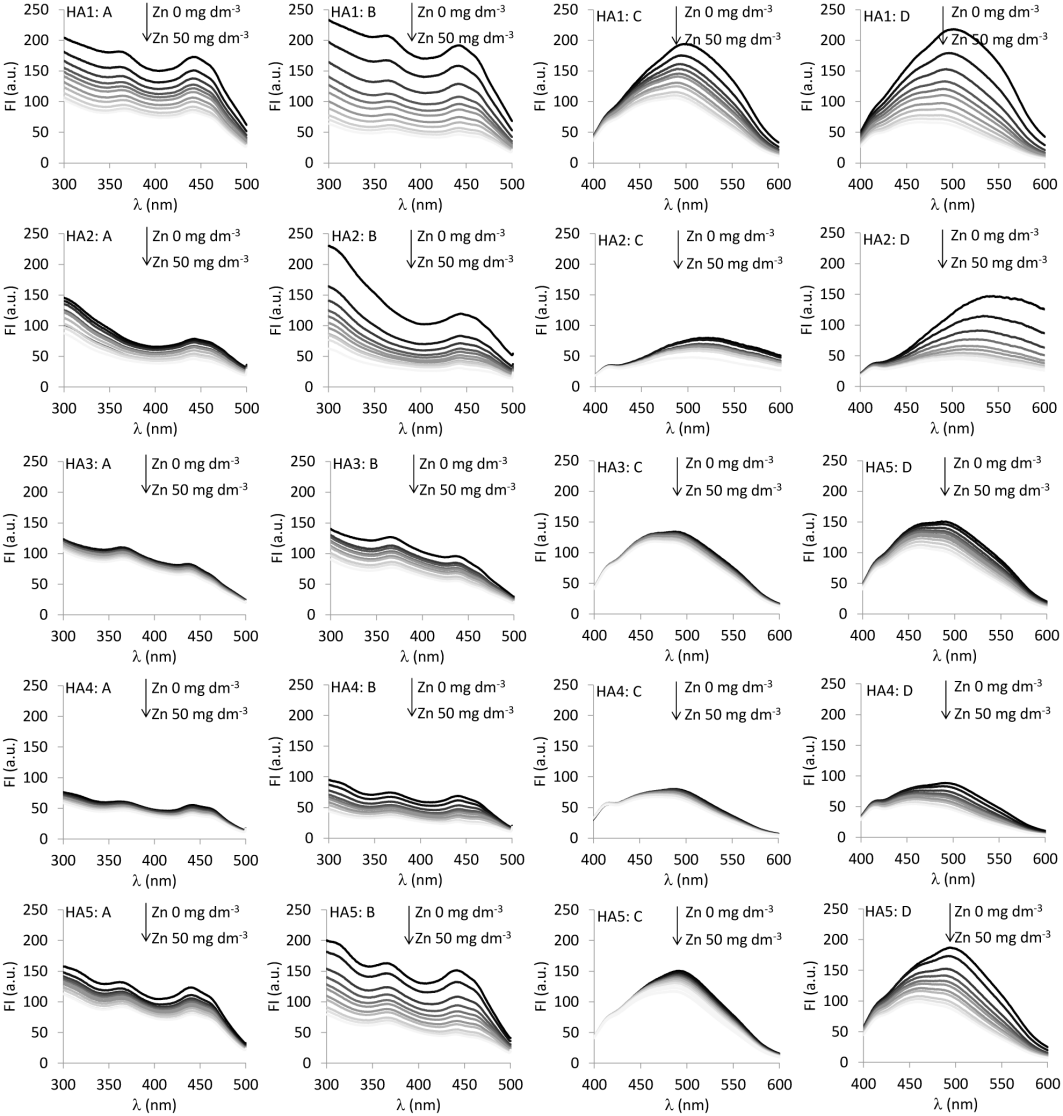
Excitation (column A—pH 5, B—pH 7) and emission (C—pH 5, D—pH 7) spectra of HAs with increasing Zn concentration.

Emission and excitation spectra of the studied HAs differ from each other both in terms of location of the peaks, as well as intensity of the maxima (see Fig 1: the highest curves are for the samples without Zn). Excitation spectra (Fig 1A and 1B columns) exhibit two maxima (one for HA2): one at ~440 nm, associated with more humified structures and one at ~360 nm, related to low molecular and less-humified fluorophores. Emission spectra (Fig 1C and 1D columns) are characterized by one broad maximum in the long wavelengths, which indicates the presence of structures with a high degree of aromaticity and with high molecular weight [27]. The increase of pH from 5 to 7 does not change the location of the main maxima; however, at the higher pH, a stronger fluorescence signal is observed both for excitation and emission maxima.

Increasing concentration of Zn(II) ions caused a quench of fluorescence in the emission and excitation spectra, which is the evidence of complexation process with the metal (Fig 1A and 1B columns—excitation, pH 5 and 7; C, D columns—emission pH 5 and 7).

Signal quenching was strong at initial, low Zn(II) concentrations, then became weaker and weaker. The FI drop was stronger at pH 7 than at pH 5, and additionally, it was the strongest for HA1 and the lowest for HA4. Additionally, emission spectra revealed the maximum shift towards lower wavelength (blue shift). The shift was higher for pH 7 than for pH 5, indicating electron rearrangements under Zn complexation.

**Fig 2:**
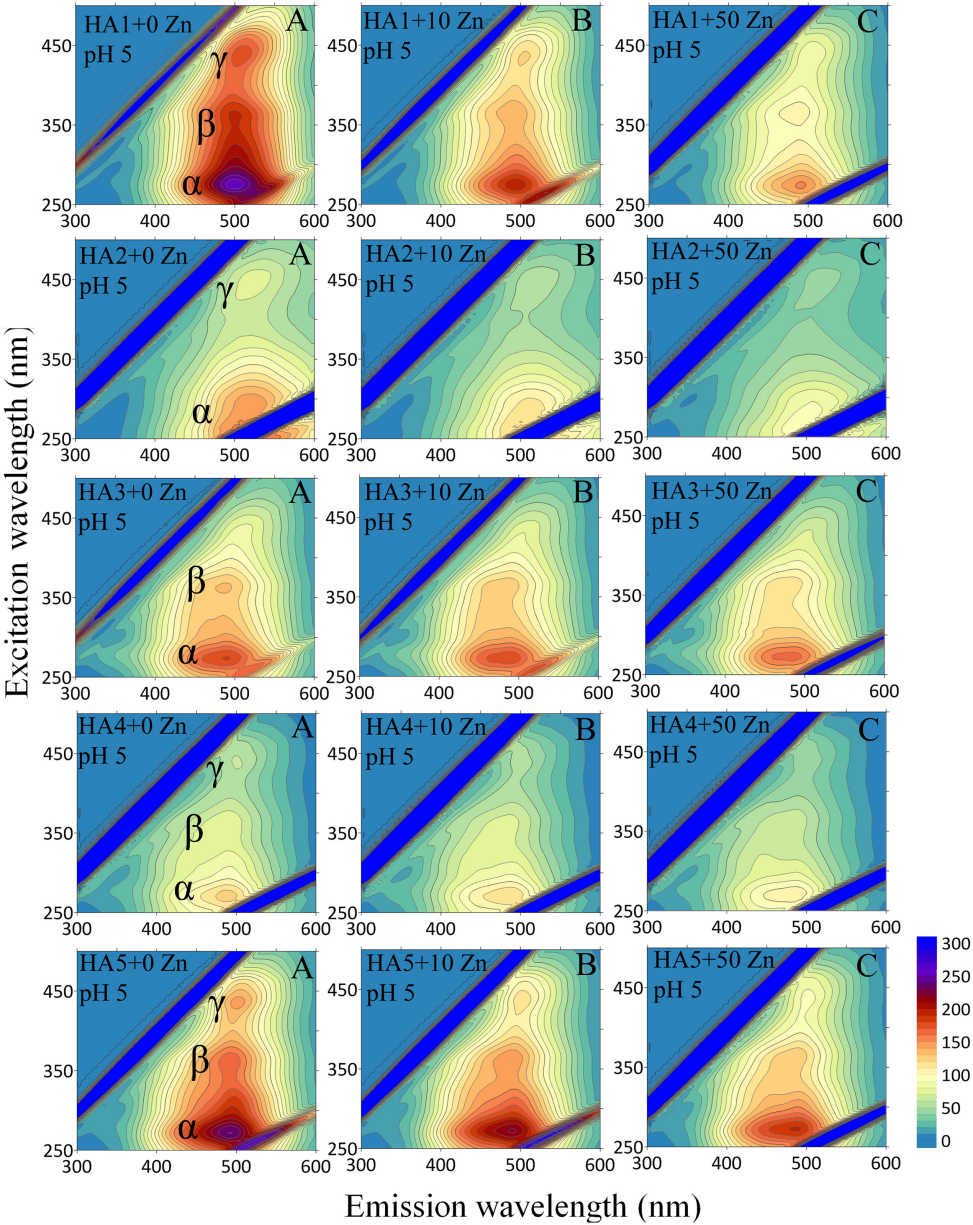
Emission-excitation (EEM) matrices for HAs and HA-Zn systems with Zn concentrations (0, 10 and 50 mg dm^-3^) at pH 5.

**Fig 3:**
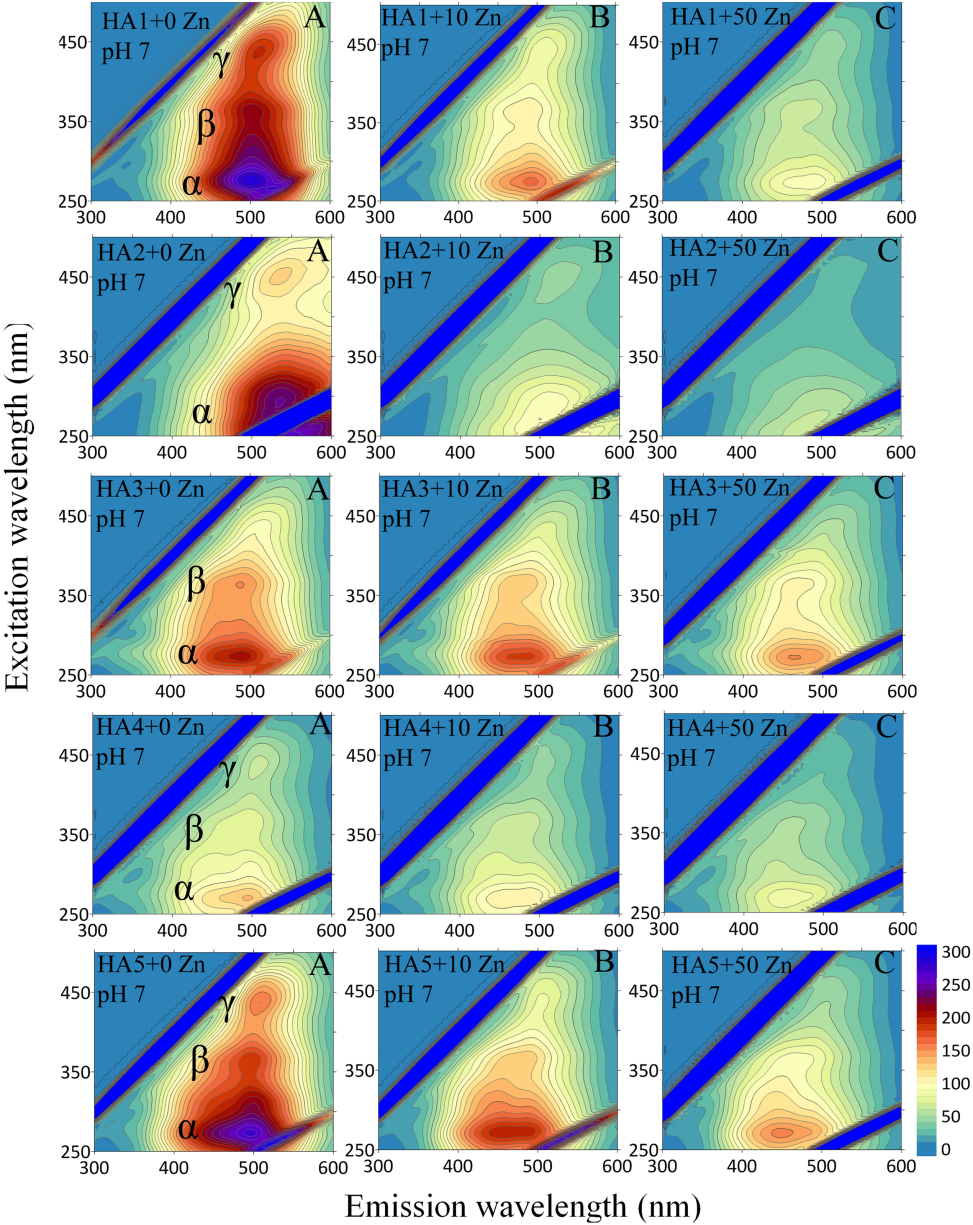
Emission-excitation (EEM) matrices for HAs and HA-Zn systems with Zn concentrations (0, 10 and 50 mg dm^-3^) at pH 7.

A more comprehensive view of HA-Zn interactions was obtained from analysis of EEM spectra. This mode of fluorescence displays the exact position of multiple maxima of fluorophore groups in HAs, as well as changes in the activity of these structures under increasing Zn(II) concentrations. Exemplary EEM matrices for the HAs, with selected Zn amounts at pH 5 and 7 are depicted in Figs 2 and 3.

In general, HA1, HA4 and HA5 reveal the presence of three fluorescence areas (marked as α, β and γ), while the HA2 and HA3 samples are characterized by only 2 areas, α and γ, and α and β, respectively. The locations of maxima for the same areas are similar for the studied HAs and are placed for α sites at excitation/emission wavelengths: 270–275 nm/490–500 nm, for β sites at 355–365 nm/485–500 nm and for γ sites at 435–450 nm/490–535 nm. The strongest FI is seen in the α-area, while the lowest one in γ areas. According to the literature, α sites are associated with the presence of simple structural components of wide heterogeneity, low-molecular weight and aromatic polycondensation. High FI can originate from electron-donating constituents, e.g., hydroxyl and metoxyl groups [28]. The β area is typical of terrestrial HAs [29] and can give this signal from weakly humified structures, simple phenols, coumarins and alkaloids [30]. The activity of γ sites can be ascribed to the presence of extended, linearly condensed aromatic rings with unsaturated bonds capable of a great degree of conjugation in large molecular-weight units with a high degree of humification [31]. In all of the areas, fluorescence signals are higher at pH 7 than at pH 5, which can be explained by a higher degree of dissociation and better spatial development of the structure of HAs at higher pH.

An increase of Zn(II) concentrations caused quenching of fluorescence at the α, β and γ sites in all HAs at pH 5 and 7, which, similarly to emission and excitation spectra, provides evidence of complexation of Zn(II) ions to different types of fluorescent binding sites on HAs [28,32]. Examples of the EEM fluorescence spectra of the HAs and their selected HA-Zn(II) systems at pH 5 and 7 are presented in Figs 2 and 3. No significant shifts of fluorescence maxima at pH 5 are observed in these particular HAs with Zn(II) addition. An increase in Zn(II) ions at pH 7 resulted in a small maximum shift (especially at the α site) towards lower wavelength (blue shift), which can demonstrate electron rearrangements and changes in chemical nature of reactions [33]

**Fig 4:**
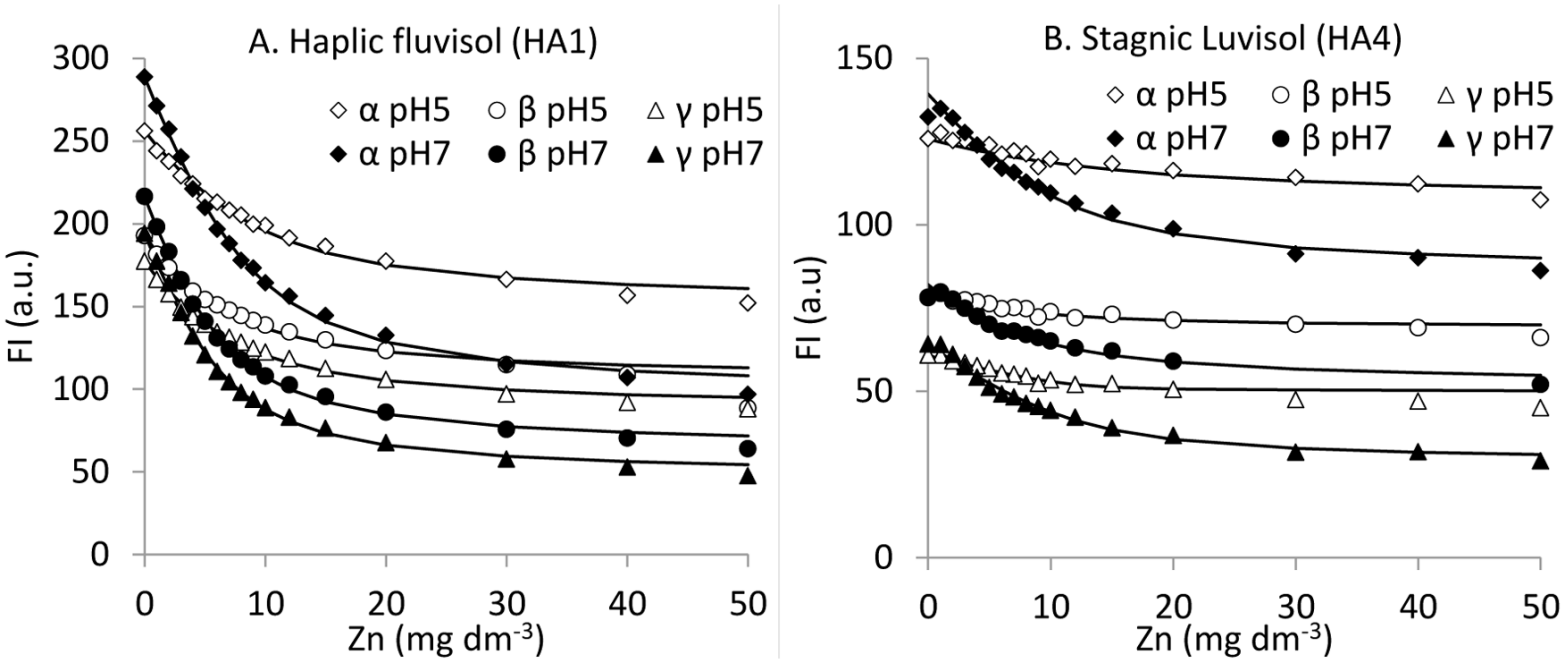
Fluorescence intensity (FI) of α, β and γ peaks of HA1 (A) and HA4 (B) determined experimentally (symbols) compared with the theoretical curves calculated from the model (solid lines) as a function of Zn(II) amount at pH 5 and 7.

FI changes under Zn(II) addition were described quantitatively by mathematical model of Ryan and Weber [26], based on a non-linear regression analysis of the quenching profiles. Fig 4a and 4b present examples of the experimental FI values (symbols) of the α, β and γ fluorophores at pH 5 and 7 as a function of Zn(II) concentration and the non-linear regressions generated by best-fitting these data with the regression model. R values exceeded 0.95 in all cases, confirming the reliability and accuracy of the model used (see also Table 3). The highest FI drop occurred at the initial, low Zn concentration, while at higher concentrations, the FI changes appear to be less and less pronounced until the Zn(II) concentration reaches a value above which no further decrease of signal is measured (see Fig 4, as well as Figure in S1 Appendix). This suggests that the maximum binding of metal ions by HA has been achieved [26].

**Table 3:** Parameters generated by fitting experimental, fluorescence data of α, β and γ sites to the model of Ryan and Weber [26], i.e., the correlation coefficients of predicted vs. measured fluorescence intensity (R), the stability constants of HA-Zn complexes (logK), and the complexing capacities (C_L_) of HAs.

	**α pH 5**				**β pH 5**				**γ pH 5**			
**HAs no**	**EEWP**	**C**_**L**_	**logK**	**R**	**EEWP**	**C**_**L**_	**logK**	**R**	**EEWP**	**C**_**L**_	**logK**	**R**
	**(nm/nm)**	**(mmol g**^**-1**^**)**			**(nm/nm)**	**(mmol g**^**-1**^**)**			**(nm/nm)**	**(mmol g**^**-1**^**)**		
HA1	275/500	2.96	4.21	0.994	360/500	1.92	4.25	0.995	440/510	2.05	4.24	0.995
HA2	270/500	0.40	4.01	0.990	n.d.	n.d.	n.d.	n.d.	445/525	0.83	4.09	0.989
HA3	275/490	4.97	3.96	0.956	365/485	2.50	4.00	0.984	n.d.	n.d.	n.d.	n.d.
HA4	270/490	3.02	3.77	0.958	355/485	1.55	4.23	0.951	435/500	4.30	4.97	0.992
HA5	270/500	0.88	4.04	0.984	360/490	2.30	3.98	0.986	440/490	3.88	4.23	0.993
	**α pH 7**				**β pH 7**				**γ pH 7**			
**HAs no**	**EEWP**	**C**_**L**_	**logK**	**R**	**EEWP**	**C**_**L**_	**logK**	**R**	**EEWP**	**C**_**L**_	**logK**	**R**
	**(nm/nm)**	**(mmol g**^**-1**^**)**			**(nm/nm)**	**(mmol g**^**-1**^**)**			**(nm/nm)**	**(mmol g**^**-1**^**)**		
HA1	275/500	3.05	4.39	0.998	360/500	2.13	4.43	0.999	435/510	2.31	4.48	0.999
HA2	270/500	0.75	4.39	0.998	n.d.	n.d.	n.d.	n.d.	450/535	0.68	4.58	0.998
HA3	275/490	5.64	4.06	0.984	365/485	2.71	4.05	0.990	n.d.	n.d.	n.d.	n.d.
HA4	270/495	3.76	4.30	0.996	365/495	2.00	4.14	0.980	435/510	4.38	4.42	0.995
HA5	270/506	2.27	4.44	0.998	365/495	2.38	4.37	0.997	435/495	3.01	4.38	0.995

n.d.—no data, EEWP—excitation-emission wavelengths pairs

Intensive quenching of FI at low Zn(II) concentrations is probably related to the existence of a high amount of unsaturated, reactive sites on HA surface, to which Zn(II) has easy access to and can be abundantly complexed. The quenching of FI at pH 7 is stronger in all cases than at pH 5, suggesting more extended HA-Zn(II) complexation at higher pH values. This result at pH 7 can be related to the high degree of dissociation of acidic functional groups responsible for complexation [21] and reduced quantity of H^+^ ions that would otherwise compete for adsorption sites. Electrostatic repulsion forces between these ionized groups energetically favor a more expanded molecular configuration, thus making binding sites readily accessible to the metal ions [34]. All the above interpretations have been confirmed by the values of the complexation capacities (C_L_) and stability constants of HA-Zn(II) complexes (logK) calculated from the model (Table 3).

High logK values confirm the stable character of binding between Zn(II) ions and HAs. The logK values listed in Table 3 are in most cases higher for pH 7 than for pH 5 and in most cases highest for the γ area. According to Plaza et al. [28], high stability of these compounds may result from the high content of acidic functional groups and other O-containing ligand groups, as well as a considerable aromatic character and high degree of humification. Aromatic carboxyl groups and adjacent phenolic groups, or two adjacent aromatic carboxyl groups are known to form highly stable ring structures with metals [35]. Phenolic structures start to dissociate at alkaline conditions so the above facts can explain higher logK values at pH 7 than at pH 5.

The values of C_L_ at α and β sites were affected by pH and were greater at pH 7 than at pH 5. For γ sites this trend was not observed. However, in the most cases the values of C_L_ were higher for γ and α areas than for β areas. HA3 showed the highest values of C_L_ within α and β areas, while HA4 showed the highest within the γ area. This fact is interesting because the above two samples exhibited different chemical properties. HA3 was characterized by the highest O/H ratio and COOH content, as well as high ω, whereas HA4 demonstrated the lowest values of OH, O/C, O and ω parameters, as well as low content of COOH. This can indicate different complexation features of fluorescence sites in relation to Zn binding: a) with marked participation of oxygen functional groups within α and β sites, and b) with lower significance of oxygen structures like COOH and OH within the γ area.

### Infrared spectroscopy

**Fig 5:**
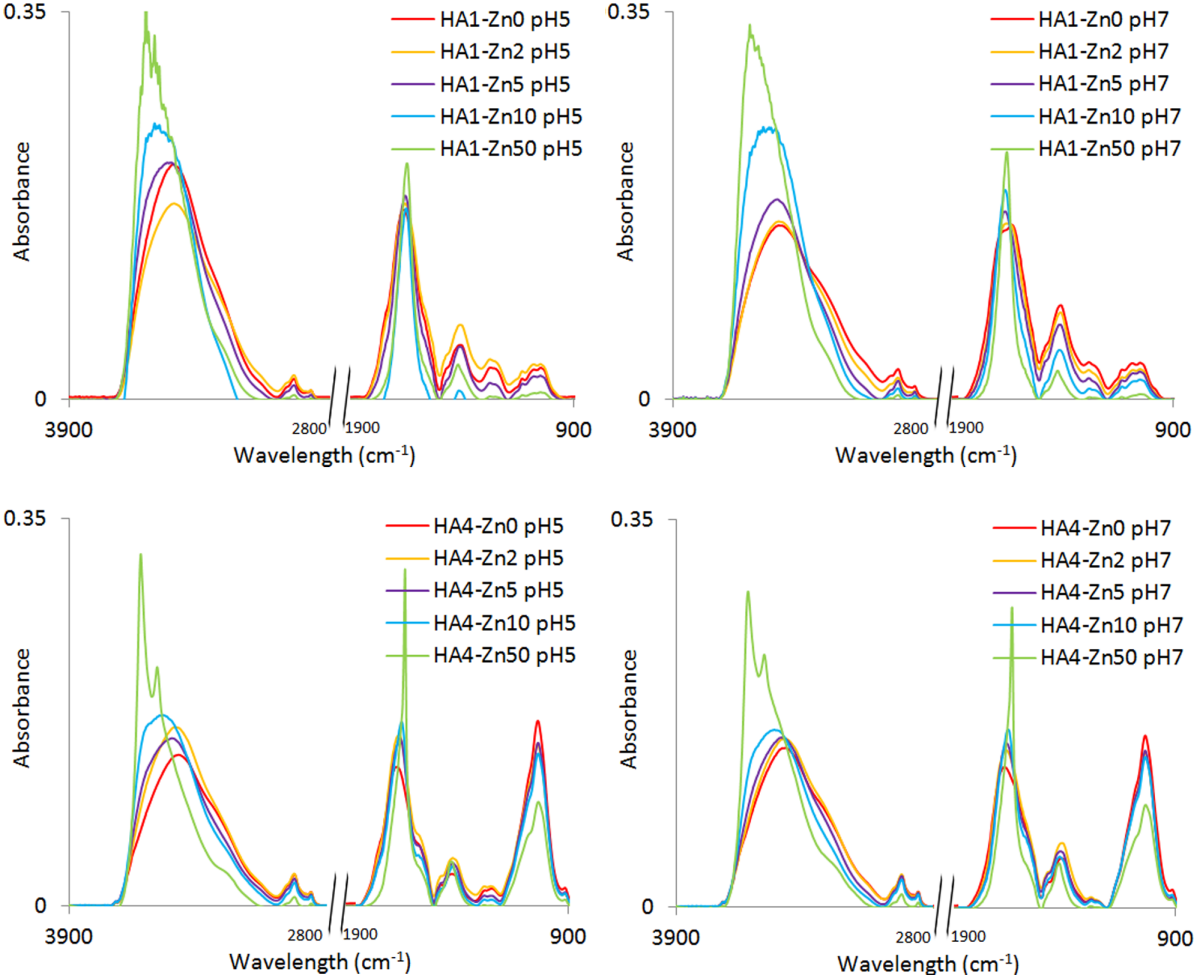
FTIR spectra of HA1 and HA4 at pH 5 and 7 with increasing Zn(II) concentrations (mg dm^-3^).

The FTIR spectra of HAs and HA-Zn complexes gave additional information about structural changes in effect of interactions with Zn(II) ions. Examples of the above spectra for selected complexes at pH 5 and 7 are presented in Fig 5. The spectra of HAs not treated with Zn(II) at pH 5 and pH 7 differed in intensities of particular bands and, to a slight degree, in location.

The intensive absorption band in the range: 3425–3450 cm^−1^ is attributed to OH stretching, the weak shoulder at ~3290 cm^−1^ to N–H and/or O–H stretching, and the double band at ~2853 cm^−1^ and at ~2925 cm^−1^ to aliphatic C–H stretching vibrations [36]. Two important bands originating from asymmetric and symmetric vibrations of C = O in carboxylate groups (COO^-^) are localized at 1598–1645 and 1387–1416 cm^−1^, respectively. The peak at 1598–1645 cm^−1^ can also be partly explained by C = C stretching in aromatic rings [5,37]. Protonated carboxylic groups (COOH) evoke the signal from asymmetric and symmetric stretching vibrations of C = O in COOH in two areas: as the shoulder at ~1710 cm^−1^and as a weak band at 1210–1267 cm^−1^, respectively [38]. The shoulder/small peak appearing at 1513–1552 cm^−1^ can be assigned to amides and C = C stretching in aromatic rings [36]. Low intensive bands at 1030–1170 cm^−1^ can be attributed to different kinds of alcohol, ether and polysaccharide groups [7]. The frequencies of the characteristic FTIR bands are presented in Table 4.

**Table 4:** Frequencies of the characteristic FTIR bands.

	HA1		HA2		HA3		HA4		HA5	
Functional groups assignment	pH 5	pH 7	pH 5	pH 7	pH 5	pH 7	pH 5	pH 7	pH 5	pH 7
	Wavelength (cm^-1^)									
OH stretching (phenols and alcohols): H-bonded OH, free OH, intermolecular bonded OH	3450	3442	3443	3443	3431	3425	3425	3425	3441	3441
N-H and O-H stretching	3291s	3288s	3301s	3301s	3288s	3295s	3294s	3288s	3291s	3304s
Aliphatic C-H asym. stretching	2927	2926	2926	2925	2932	2929	2925	2924	2925	2923
Aliphatic C-H sym. stretching	2854	2854	2854	2854	2853	2853	2853	2853	2853	2852
C = O asym. stretching in COOH	1710s	-	1710s	-	1714s	-	1710s	-	1710s	-
C = O asym. stretching in COO^-^, C = C stretching in aromatic rings	1635	1598	1633	1627	1645	1599	1644	1640	1629	1630
Amide bands, C = C stretching in aromatic rings	1545s	-	-	-	1517	1513s	1552s	-	-	-
C-H aliphatic bending, C = C aromatic stretching	1443s	1450s	-	1447s	1447s	1443s	1450s	1450s	1467s	1447s
C = O sym. stretching in COO^-^, C-H aliphatic sym. stretching	1387	1387	1404	1401	1416	1387	1404	1395	1389	1388
C = O sym. stretching in COOH	1262	1266	1216s	1210s	1238	1264	1233	1266	1266	1267
OH tertiary alcohol	-	-	-	-	1170	1160s	-	-	-	-
OH secondary alcohol, CxHy aliphatic and cyclic	1125	1122	1107	1107	1125	1123	-	-	1117s	1111s
C-O-C aliphatic ethers	1073	1078	1108	1105	1084	1084	-	-	1075	1078
OH primary alcoholic, CO polysaccharide stretching	1044	1045	1034	1032	1047	1045	1033	1032	1044s	-

A comparison of FTIR spectra of HAs and HA-Zn complexes provides information about changes in functional groups, as well as data on modes of complexes. Significant changes under Zn influence are observed for the OH band at 3425–3450 cm^−1^. Intensity of this band increases with Zn concentration for HA2, HA4 and HA1 (only at pH 7) and initially decreases and then increases with Zn amount for the HA3, HA5, HA1 (only at pH 5). A drop of absorption of this band at low Zn amounts can result from binding of the metal by OH groups [34], while an increase from the presence of hydration water and production of aqua-complexes [5]. An increase in Zn concentration shifts maximum of the band towards higher frequencies, indicating a change in the coordination sphere of the complex. Zn(II) binding by OH groups has been also confirmed by a drop of absorbance of the bands attributed to different kinds of OH groups localized within area of 1030–1170 cm^−1^.

The HA-Zn(II) complexes are also formed with COOH contribution, visible as a decrease in the absorption shoulder at ~1710 cm^−1^ (asymm. COOH) and at 1210–1267 cm^−1^ (symm. COOH), and simultaneously, as an increase in the bands at 1598–1645 (asymm. COO^-^) and at 1387–1416 cm^−1^ (symm. COO^-^). Due to the possible minor influence of various other structures, the changes described above have not always been proportional to Zn concentrations. Proton exchange in COOH is stronger at pH 5, due to a higher amount of non-dissociated carboxyl groups. However, even at the highest Zn amount in complexes, some small part of the band at about 1260 cm^-1^ does not disappear, especially at pH 5, suggesting that not all COOH groups are involved in Zn binding, which could result from steric effects. The possibility of coordination by nitrogen atoms should also be considered due to changes in absorption shoulders at the region of 1513–1552 cm^-1^. That region, however, can be also affected by the signal from vibrations in aromatic rings [36]. An increase in Zn(II) concentration at pH 7 also causes a shift of the maximum of the asymm. COO^-^ band toward lower frequencies. This process provides evidence of a rearrangement of the molecules to another type of coordination [34]. At pH 5 (except for HA4) the above shifts were not as pronounced for the complexes.

**Fig 6:**
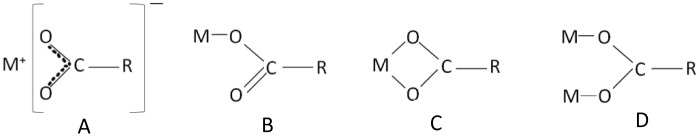
Modes of metal binding by carboxylate ligands: A—ionic or uncoordinated forms, B—unidentate complexes, C—bidentate chelates, D—bidentate bridging coordination.

**Fig 7:**
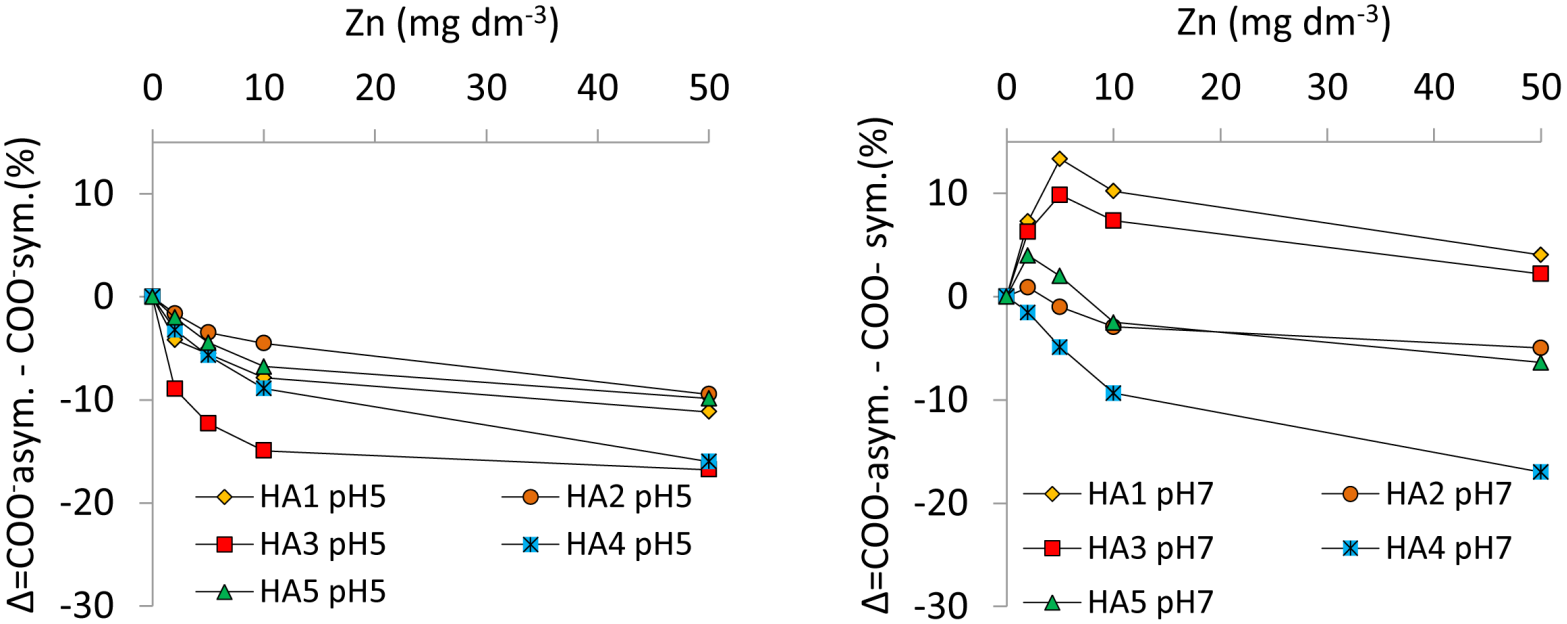
Percentage changes of (ΔCOO-) in HA-Zn complexes at pH 5 and 7 in relation to ΔCOONa in ionic form.

Increasing Zn(II) concentrations in the complexes with HAs also results in changes in the distance between the symmetric and asymmetric COO^-^ bands. According to the literature, analysis of this distance (ΔCOO^-^) in relation to the distance in ionic form (for example, in sodium salt: ΔCOONa) can be useful in establishing the bonding mode and some conclusions about stability of complexes [39]. In this paper, the values of (ΔCOO^-^) were calculated for the HA-Zn complexes with various metal concentrations and then compared with the values of (ΔCOONa) for studied HAs. On this basis different kinds of Zn interactions with ligands have been considered (Fig 6), including: (A) ionic or uncoordinated forms, (B) unidentate complexes, (C) bidentate chelates, and (D) bidentate bridging coordination. The obtained results show that the chemical character of the binding changes with an increase of Zn concentration and with pH. Fig 7 depicts the changes of (ΔCOO^-^) with Zn concentration in complexes at pH 5 and 7.

HA-Zn preparations at pH 5 result in lower **(**ΔCOO^-^) values compared to the ionic form. Moreover, (ΔCOO^-^) decreases with an increase in Zn concentration. The drop is stronger at low Zn concentrations and then it becomes lower and lower until a plateau is reached for the highest Zn concentrations. The differences (ΔCOO^-^) for HA1, HA2 and HA5 complexes remain close to relevant ionic values of (ΔCOO^-^) over the whole Zn concentration range (max drop ~10%), so that bidentate bridging coordination can be a dominant mechanism for Zn binding in these samples. The complexes of HA3 and HA4 exhibit much lower values of (ΔCOO^-^), especially at higher Zn concentrations, suggesting bidentate chelation.

Analysis of (ΔCOO^-^) in HA-Zn complexes at pH 7 shows initial increase of this parameter in relation to (ΔCOO^-^) of ionic form, what suggests the formation of unidentate complexes at low Zn concentrations. Higher Zn concentration causes a decrease of (ΔCOO^-^), which indicates an increasing role of bidentate bridging coordination. At low Zn concentrations, population of unidentate complexes is the most pronounced for HA1 and HA3 (the highest increase of (ΔCOO^-^)). It should be noticed that these HAs are characterized by the highest ω values and a high content of COOH+OH groups, that can take part in the formation of the bindings with Zn. Such trends in the changes of (ΔCOO^-^) are not so strongly pronounced in the case of HA2 and HA5, suggesting only bidentate bridging coordination. Only for HA4 do the values of (ΔCOO^-^) not demonstrate the initial step typical for unidentate complexes. In the latter case a strong, continuous decrease of (ΔCOO^-^) is observed, indicating a dominant influence of bidentate bridging and then bidentate chelation processes. The above observations suggest differences in complexation at pH 5 and 7, as well as, in some cases, the existence of a two-step mechanism that depends on Zn concentration. At lower pH, the dominant process is bidentate bridging coordination and at higher Zn concentrations, bidentate chelation. At higher pH (except for HA4) the dominant process is unidentate complexation and at higher Zn concentrations bidentate bridging.

### Proton releasing study

**Fig 8:**
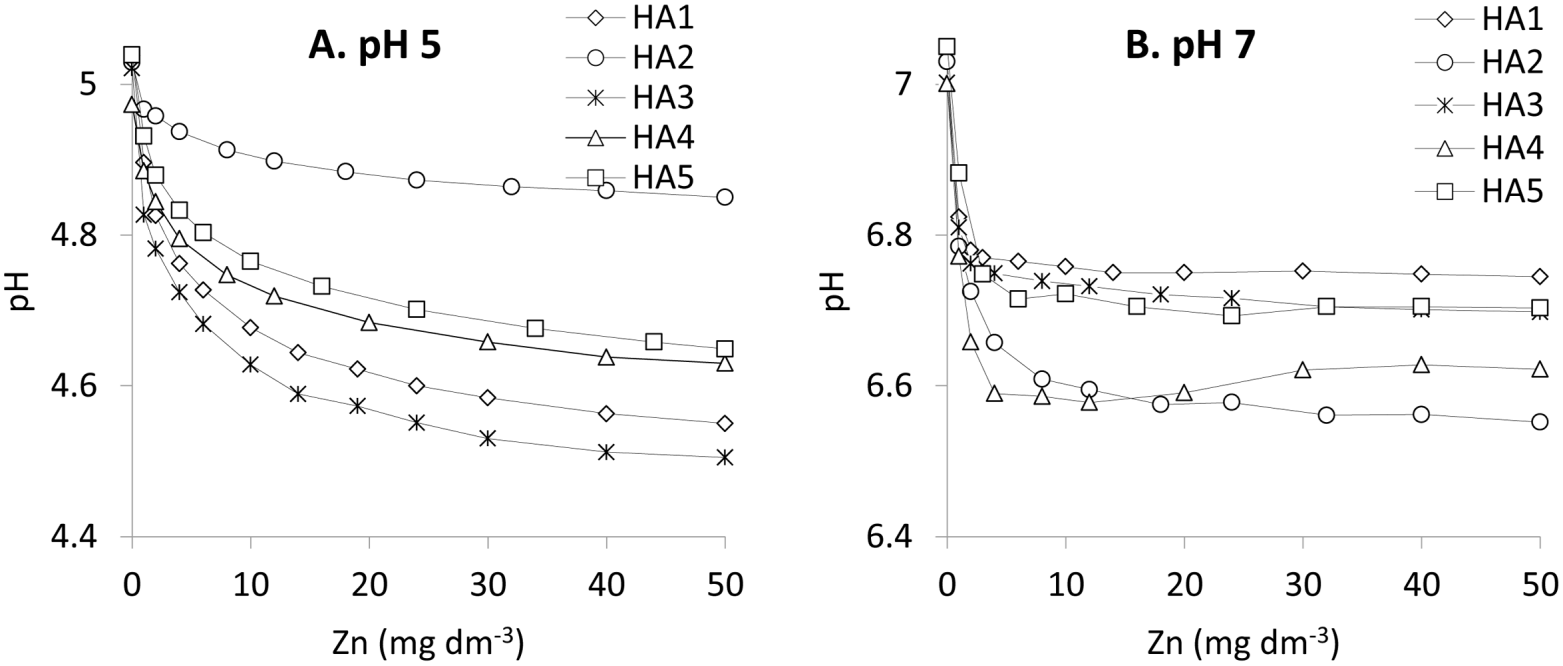
Drop of pH in solutions of the HAs from the values 5 (A) and 7 (B) as function of increasing Zn(II) concentration.

A pH drop in HAs solutions under increasing Zn(II) concentration provides evidence of proton exchange and chemical interactions between non-dissociated functional groups and metal ions [35]. The curves describing the dependence of pH vs. Zn(II) concentrations are similar in shape to the curves fit to experimental data from fluorescence using the Ryan and Weber model, cf. Fig 8.

Starting from pH 5 and also 7 the drop is the strongest at low Zn concentration, while at higher Zn concentrations proton exchange is weaker and weaker until the Zn(II) concentration attains a value above which further pH decrease is insignificant. Such behavior is observed due to the presence of multiple available functional groups, which are able to bind easily to low amount of Zn(II) ions. At higher Zn concentrations, some steric effects originating from adjacent, occupied functional groups can weaken activity of neighboring free groups [21,28]. At pH 7 this plateau is attained at lower Zn concentrations than at pH 5. The biggest pH drop, starting from pH 5, was measured for HA3 and the smallest for HA2. The drop of pH from 7 is the highest for HA2 and the lowest for HA1.

The value of the pH drop at pH 7 is not greater than at pH 5. It should be emphasized, however, that, in general, the pH drop is related to protonated groups, so it does not include interactions of the metal with groups already dissociated below pH 5 or 7. In consequence, a decrease of pH at pH 7 is due mostly to protonated groups like OH and for this reason it can be similar or even lower than for pH 5, where values of pH drop comes from both part of the undissociated COOH and all OH groups.

### Relationship between parameters describing Zn-HAs interactions and HAs properties

Statistical analysis of obtained results points to significant influence of some chemical properties of HAs on interactions of HAs with Zn(II). Matrix of correlation coefficients is presented in Table 5.

**Table 5:** Coefficients of Person’s correlation calculated between selected chemical properties of HAs and the complexation capacities of HAs (C_L_), the stability constants of HA-Zn(II) complexes (logK) and pH drop at pH 5 and 7. Bold digits highlight statistically significant relationships.

	O	H/C	O/H	O/C	C/N	ω	COOH	OH	COOH +OH	E4/E6	E2/E6	ΔlogK
**pH 5**												
**C**_**L**_ **(α)**	0.23	-0.47	0.75	-0.05	0.03	0.56	0.47	-0.35	0.25	0.85**	0.86**	0.93*
**C**_**L**_ **(β)**	0.93**	0.74	0.73	0.88	-0.56	0.67	0.99*	0.17	0.84	0.43	0.48	0.40
**C**_**L**_ **(γ)**	0.33	-0.02	0.43	0.18	0.80	-0.04	0.56	0.00	0.39	0.97*	0.87	0.66
**logK (α)**	0.39	0.36	0.31	0.40	-0.89*	0.65	0.20	0.80	0.46	-0.45	-0.40	-0.34
**logK (β)**	-0.86	-0.84	-0.32	-0.88	0.13	-0.24	-0.92**	-0.04	-0.72	-0.46	-0.44	-0.28
**logK (γ)**	-0.39	-0.66	0.07	-0.55	0.89	-0.39	-0.12	-0.57	-0.29	0.86	0.97*	0.94*
**pH drop**	0.33	-0.28	0.68	0.07	0.52	0.34	0.54	0.08	0.46	0.66	0.44	0.47
**pH 7**												
**C**_**L**_ **(α)**	0.38	-0.31	0.78	0.11	0.17	0.53	0.62	-0.32	0.38	0.95*	0.92*	0.95*
**C**_**L**_ **(β)**	0.79	0.55	0.71	0.72	-0.42	0.57	0.92**	-0.13	0.64	0.68	0.72	0.65
**C**_**L**_ **(γ)**	0.13	-0.31	0.47	-0.09	0.80	-0.02	0.40	-0.11	0.23	1.00*	0.95*	0.86
**logK (α)**	-0.19	0.27	-0.48	-0.02	-0.04	-0.33	-0.40	0.64	-0.08	-0.87**	-0.97*	-0.98*
**logK (β)**	0.20	0.33	-0.01	0.24	-0.43	0.26	-0.07	0.96*	0.42	-0.95*	-0.91*	-0.88
**logK (γ)**	-0.62	-0.21	-0.64	-0.46	-0.55	-0.26	-0.81	-0.34	-0.68	-0.86	-0.67	-0.48
**pH drop**	-0.23	0.13	-0.52	-0.11	0.84**	-0.76	-0.23	-0.17	-0.25	-0.10	-0.26	-0.37

*p = 0.05;

**p = 0.1

At both pH values, 5 and 7, the values of C_L_ for α area demonstrate high and positive correlations with E4/E6, E2/E6 and ΔlogK. Thus, the amount of complexed metal increases along with a decrease of humification and degree of aromaticity of the HA structure, as well as with a decrease in molecular weight. Similar relationships were found for the γ site, however at pH 5 despite of high R values for E2/E6 and ΔlogK, only E4/E6 correlates significantly with C_L_. Positive and significant correlations were also found between C_L_ of β sites and COOH content at pH 5 and 7, as well as O content at pH 5. The remaining C_L_ correlations, which related to “oxygen” parameters of HAs, are insignificant. However it should be noticed that in case of each C_L_, correlations with COOH content as well as O/H and ω parameters are also positive and in most cases higher for α and β than γ sites. This may suggest higher significance of oxygen groups in Zn complexation by α and β than γ structures, however such cautious supposition requires additional studies.

LogK changes differently with properties of HAs. At pH 5 this parameter decreases with an increase of C/N for α area and with an increase of COOH content for β sites; this parameter increases with an increase of E2/E6 and ΔlogK for γ fluorescence sites. At pH 7, logK of the complexes of α and β sites evidently decrease with increasing values of E4/E6 and ΔlogK. Long, expanded structures—typical for high values of E4/6 E2/E6 and ΔlogK—facilitate complexation, however the formation of the most stabile bindings is hampered by the longer distances between neighbored functional groups (in general, simple complexes formed from monodentate ligands are not as stable as chelates formed from closely located functional groups, e.g., connected to aromatic ring). No significant relations were found for the γ site suggesting a multifactor impact on the values of logK. The pH drop did not reveal many statistically significant dependencies, indicating the possibility of complexation not only with proton exchange but also with interactions with dissociated groups and donor atoms like nitrogen.

## Conclusions

The application of fluorescence spectroscopy including excitation, emission and 3D spectra demonstrates complex nature of interactions between HAs and Zn(II) ions. The studies have revealed the presence of a few areas of HAs structures responsible for complexing with Zn(II). The complexation process at α (simple structural components of low-molecular weight and aromatic polycondensation) and β areas (weakly humified structures with simple phenols, coumarins and alkaloids) was stronger at pH 7 than 5, which was demonstrated by the complexation capacities. This trend was not observed for γ sites (extended, linearly condensed aromatic rings with unsaturated bonds, structures with large molecular weight and highly humified). Stronger Zn(II) complexation at higher pH was connected with a higher dissociation degree of functional groups, as well as with greater expansion of the structure, minimizing steric effects.

The amount of metal complexed at pH 5 and 7 by the structures from α and γ areas increased significantly together with a decrease of humification and aromaticity degree as well as with the molecular weight of humic acids. This was in contrast to the β structural area, where complexation capacity significantly increased only with an increase of the amount of carboxylic groups. At pH 7 the chemical nature of the complexes changed with Zn concentration, which was revealed by shifts of signals maxima both in fluorescence and FTIR analysis. Binding of Zn ions was most intensive at initial, low metal concentrations, related to the existence of a high amount of free, unsaturated reactive sites on the HA surfaces to which metal ions have an easy access. In the most cases, the stability of the complexes was higher for pH 7 than for pH 5 and at each pH it was the highest for the γ fluorescence area, which was confirmed by the calculated logK values. For complexes formed at pH 5 this parameter decreased with an increase of C/N for α area and with an increase of COOH content for β sites; this parameter increased with a decrease of humification of HAs for γ structures. At pH 7 the stability of the complexes within α and β areas decreased with a decrease of HAs humification.

Analysis of changes in intensities of FTIR bands showed that HA-Zn(II) interactions were strongly influenced by the presence of COOH and OH functional groups, aromatic and nitrogen-containing structures, as well as by the formation of aqua-complexes. Even at the highest Zn concentrations, not all COOH groups were occupied by Zn, which probably resulted from steric effects. Analysis of the FTIR spectra also revealed the presence of different modes of complexation depending on Zn concentration and pH. For pH 5, the most humified HAs tended to show bidentate bridging coordination at all Zn concentrations, but in the case of the least humified HAs, Zn caused bidentate bridging coordination at low Zn concentrations, with a tendency to bidentate chelation processes at the highest Zn concentrations. At pH 7, low Zn concentrations caused the formation of unidentate complexes, while higher Zn doses, bidentate bridging. Such processes were noticed for HAs characterized by a high degree of oxidation and oxygen functional group content. HAs with lower content of these structures displayed only bidentate bridging coordination or even bidentate chelation for the HA with the highest carbon content and the lowest H/C and O/C ratios.

Humic acids partly interacted with Zn (II) ions in terms of proton exchange, which was confirmed by potentiometric and FTIR analyses. For pH 7, the equilibrium state of the above processes was attained at lower Zn concentrations than for pH 5, which was due to lower number of protonated groups at pH 7, as well as better spatial development of structures of humic acids with higher susceptibility to Zn binding. At pH 5 the most intensive protons exchange was related to the HA characterized by the lowest humification degree, as well as by being rich in oxygen structures, which was confirmed by the values of O/H ratio, ω parameter, and the amount of COOH and COOH+OH content. The lowest proton exchange occurred in the most humified humic acid (with the lowest values of E4/E6, Δlog K and E2/E6). In spite of the above findings, no significant relationships were found for all groups of studied humic acids, indicating the possibility of complexation not only with proton exchange but also by interactions with dissociated groups and donor atoms like nitrogen. At pH 7, statistical analysis also did not show any significant correlations between the proton exchange and the properties of HAs.

To summarize, the current study has shown significant differences in the mechanisms of interactions between humic acids and Zn ions. Both Zn concentration and pH demonstrated significant influence on the above processes. Statistical analysis also revealed significant impact of some properties of HAs on complexation reactions with zinc ions. Understanding these relationships can be helpful in prediction of the ecological effects of adding Zn as a soil nutrient or in preparation of optimal Zn-humic acid fertilizers.

## Supporting Information

S1 Appendix Model curves (solid lines) fit to experimental data from fluorescence (symbols). A—HA2, B—HA3, C—HA5; α, β, γ—fluorescence binding sites.
(TIF)
