# Supplementary figures and images for: Interactions of Zn(II) Ions with Humic Acids Isolated from Various Type of Soils. Effect of pH, Zn Concentrations and Humic Acids Chemical Properties
Source: PLoS One. 2016 Apr 14;11(4):e0153626. doi: 10.1371/journal.pone.0153626 (PMC4831678; doi:10.1371/journal.pone.0153626)

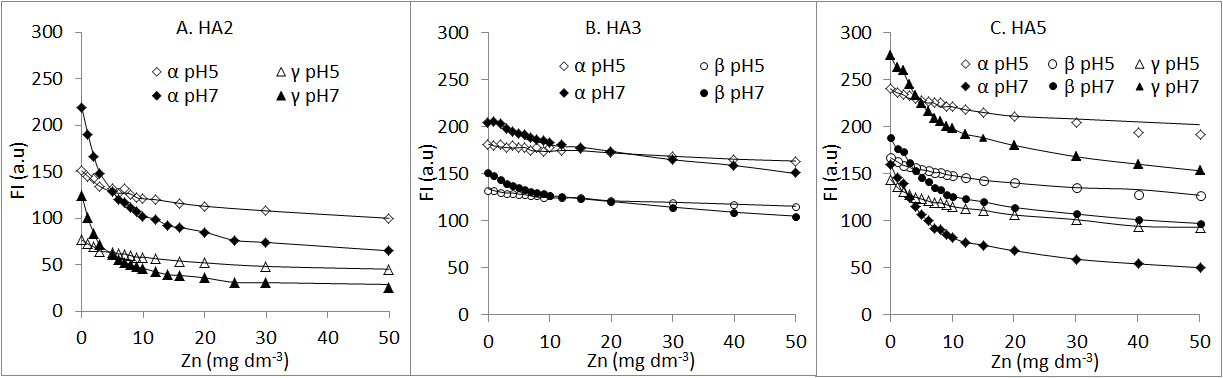

Supplement: S1 Appendix — A—HA2, B—HA3, C—HA5; α, β, γ—fluorescence binding sites. (TIF) [file pone.0153626.s001.tif]
